# Efficiency of moderately hypofractionated radiotherapy in NSCLC cell model

**DOI:** 10.3389/fonc.2024.1293745

**Published:** 2024-04-24

**Authors:** Marcus Lüdeking, Katharina Konzok, Dhanya Ramachandran, Sinja Grosche, Hans Christiansen, Roland Merten, Christoph Henkenberens, Natalia V. Bogdanova

**Affiliations:** ^1^ Radiation Oncology Research Unit, Hannover Medical School, Hannover, Germany; ^2^ Gynaecology Research Unit, Hannover Medical School, Hannover, Germany; ^3^ Radiation Oncology, Hannover Medical School, Hannover, Hannover, Germany; ^4^ Radiation Oncology, Dorothea Christiane Erxleben Clinic, Wernigerode, Germany

**Keywords:** moderated hypofractionated radiotherapy, non-small cell lung carcinoma, irradiation, cell culture model, *in vitro* study

## Abstract

**Background:**

The current standard of radiotherapy for inoperable locally advanced NSCLCs with single fraction doses of 2.0 Gy, results in poor outcomes. Several fractionation schedules have been explored that developed over the past decades to increasingly more hypofractionated treatments. Moderate hypofractionated radiotherapy, as an alternative treatment, has gained clinical importance due to shorter duration and higher patient convenience. However, clinical trials show controversial results, adding to the need for pre-clinical radiobiological studies of this schedule.

**Methods:**

We examined in comparative analysis the efficiency of moderate hypofractionation and normofractionation in four different NSCLC cell lines and fibroblasts using several molecular-biological approaches. Cells were daily irradiated with 24x2.75 Gy (moderate hypofractionation) or with 30x2 Gy (normofractionation), imitating the clinical situation. Proliferation and growth rate via direct counting of cell numbers, MTT assay and measurements of DNA-synthesizing cells (EdU assay), DNA repair efficiency via immunocytochemical staining of residual γH2AX/53BP1 foci and cell surviving via clonogenic assay (CSA) were experimentally evaluated.

**Results:**

Overall, the four tumor cell lines and fibroblasts showed different sensitivity to both radiation regimes, indicating cell specificity of the effect. The absolute cell numbers and the CSA revealed significant differences between schedules (P < 0.0001 for all employed cell lines and both assays) with a stronger effect of moderate hypofractionation.

**Conclusion:**

Our results provide evidence for the similar effectiveness and toxicity of both regimes, with some favorable evidence towards a moderate hypofractionation. This indicates that increasing the dose per fraction may improve patient survival and therapy outcomes.

## Introduction

Non-small cell lung carcinoma (NSCLC) belongs to the most common type of lung cancer and cause of cancer-related death (1) with a 5-year survival rate of about 20% (2). The standard of treatment for inoperable locally advanced NSCLCs is conventionally normofractionated radiotherapy (RT) with a single dose of 2.0 Gray (Gy) and total doses of 60-66 Gy with concurrent platinum-based chemotherapy followed by checkpoint inhibition for patients with positive PD-L1 Status≥1% (3). The aim of concurrent chemotherapy is radio sensitizing the tumor cells with cytostatic drugs via interaction with the DNA. This results in a synergistic effect of cytostatics and irradiation (IR). Some patients who cannot tolerate concurrent radio chemotherapy or refuse chemotherapy receive RT alone. Standard normofractionated RT (NormRT) with single fraction doses of 2.0 Gy results in poor outcomes with 5-year survival rates below 10% (4). Testing of dose-escalating schedule showed its association with an increased risk of esophagitis and other toxicities as severe radiogenic pneumonitis without significantly improving the clinical results (5–7), limiting the acceptance for clinical use. The failure of NormRT is associated with tumor heterogeneity and radiation-stimulated radio resistance. Accelerated dose per fraction protocols may open a therapeutic window for radiation dose optimization in NSCLC, since a small dose per fraction RT may select for radioresistant tumor cells, while hypofractionation may sensitize tumor cells to subsequent irradiation (8). Therefore, stereotactic ablative radiotherapy (SBRT) for small tumors with a few high dose fractions or moderate hypofractionated radiotherapy (HypoRT) for patients with locally advanced NSCLC, have recently gained clinical importance as alternative RT treatment (9–11). HypoRT offers a shorter treatment duration, and is, therefore, more convenient for patients with lung cancer than NormRT (12, 13), and may allow better outcomes due to improved inhibition of cancer cell repopulation via increased biologically effective doses. However, large randomized trials have not yet been conducted and there is some concern about clinical outcomes, adding the need to evaluate toxicities and loco-regional tumor control of this regime.

We aimed to investigate the efficacy of HypoRT during daily irradiation (mimicking clinical situation), and to compare the effects in contrast to NormRT in NSCLC cell-lines and healthy fibroblasts, using different molecular-biological approaches for studying radiation-induced effects.

## Materials and methods

### Cell lines

We employed the Bj5Ta fibroblasts from a healthy donor and four commonly used in basic and drug discovery research NSCLC cell-lines: A549, Nci-H522, Nci-H1650 and Nci-H1975. Employed cell-lines were obtained from the ATCC (American Type Culture Collection). BJ5ta is an hTERT-immortalized fibroblast cell-line. A549 is adenocarcinoma arising in alveolar basal epithelial cells and can be grown as adherent or in suspension. In our experimental settings, A549 cells were grown as adherent and cultured as Bj5Ta cells in DMEM (Dulbecco′s Modified Eagle′s Medium) with 500 U/ml penicillin, 2 mM L-glutamine, 0.5 mg/ml streptomycin and 10-12% FCS. Other NSCLC cell-lines are from epithelial morphology and were cultured in Gibco RPMI 1640 medium with the same supplements as above. All employed cells were maintained in a humidified 5% CO2 atmosphere at 37°C and irradiated every day until the appropriate total dose was reached: 66 Gy for HypoRT or 60 Gy for NormRT, respectively. Dead cells were eliminated by medium replacement every day. Cells were seeded in a fixed number of 9x10^5^cells per flask 48h prior to the first IR treatment.

### Experimental schedule for x-ray irradiation

Irradiation was applied using 6MVX-Photon-Beam of a SynergyTM linear accelerator (Elekta AB, Stockholm, Sweden). We selected two irradiation protocols from clinical practice, following the standard irradiation schedules used in hospitals, including our own. For normofractionation, the international standard of 60 Gy in 2 Gy daily fractions, and for moderate hypofractionation, 66 Gy in daily fractions of 2.75 Gy, were applied, as suggested in two consecutive EORTC trials (14, 15). Assuming that α/β -ratio for NSCLC cells is 10 Gy and for fibroblasts is 3 Gy, the corresponding biological effective dose (BED) equivalents for the utilized schedules according to the basic LQ (linear quadratic) model are as follows: BED_10_ (NormRT) – 72 Gy; BED_3_ (NormRT) – 100 Gy; BED_10_ (HypoRT) – 84.5 Gy; BED_3_ (HypoRT) – 126.5 Gy. During daily irradiation at a dose of 2 Gy (NormRT) per fraction or 2.75 Gy (HypoRT), the cells were kept warm at about 37°C with warm-pads to obtain a setting matching the clinical procedure. For both irradiation regimes, the dose/rate of 535 MU/min, field size of 40 x 40 cm and distance of 110 cm were used. For NormRT, 227 MU and for HypoRT, 303 MU were applied, respectively.

Each experiment for every cell-line, included non-irradiated matched controls (untreated, UNT). To perform proliferation analysis by MTT assay or via direct counts of cell numbers, the cells were sampled on every third irradiation day (16). Immunocytochemical staining (ICC) was conducted on irradiation days 3, 15 and 24 for both irradiation regimes and for A549 and Bj5Ta cells on day 30 for the NormRT protocol (16). A modified clonogenic assay or colony formation assay (CFA), as described in (16) was performed on irradiation days 24 (NormRT and HypoRT) and on irradiation days 30 (NormRT). For both irradiation regimes on irradiation day 24, and for NormRT on irradiation day 30, EdU incorporation assay (DNA synthesis–based cell proliferation) was applied, but only for A459 and Bj5Ta cells, since A459 cells had the highest survival rate and Bj5Ta cells were used as a non-cancer control.

### Proliferation assays

Cell proliferation was evaluated based on three different methods: direct counting of cell numbers, MTT assay and measurement of DNA-synthesizing cells (EdU assay, only for A459 and Bj5Ta cells).

Direct counts of cell numbers were performed from double sampling, using two counting approaches: manual and automated, as described in (16). Absolute cell numbers were calculated from two combined scores, each comprising of two independent values from manual and automated counting (a total of 4 data points were combined into an average score).

To measure growth inhibition or the cytotoxicity of both irradiation regimes, metabolic activity was measured by MTT assay as described in (16). The absorbance of the samples (OD values on the y-axis) was plotted versus experimental day (on the x-axis), and treated (irradiated) cells were compared to untreated matched cell cultures as described in (16).

DNA synthesis–based cell proliferation for A459 and Bj5Ta cells at irradiation day 24 was measured by 5-ethynyl-2’-deoxyuridine (EdU) incorporation using Click-iT® EdU Imaging Kit (Invitrogen) as described in (16). Leica DMI6000B microscope with 20x objective and a 1.6x artificial zoom was used for the visualization.

### Immunocytochemistry: fluorescent staining procedure and analysis

To perform ICC on experimental days 3, 15 and 24, the cells (in technical duplicates) were spotted on non-coated sterilized coverslips onto the wells of a 24-well plate directly after irradiation and placed in the incubator for a further 24h at +37°C and 5% CO2. The cells were fixed and fluorescently stained 24 hours after each defined irradiation day as described in (16).

We counted ‘residual foci’ that were not directly induced after irradiation. Residual foci were analysed in several areas of every duplicate. Leica DMI6000B microscope with 63x objective and a 1.6x artificial zoom was used, as described in (16). For every slide, minimum of 50 cells were visualized and each cell with at least one focus was included in the quantification analysis as a responsive one. We evaluated the number of foci per cell and the average result from two technical duplicates is presented in the results section.

### Clonogenic assay

Modified CFA, as described in (16), was performed to assess cell reproductive death after IR. We evaluated the survival ability of cells that remained after the total dose of HypoRT or NormRT was achieved, Each cell-line was seeded with a defined number of cells (**Table 1**) in duplicates after irradiation day 24 (NormRT and HypoRT) and irradiation day 30 (NormRT only). In parallel UNT matched controls for every cell-line were seeded in technical triplicates (**Table 1**). Every two days, the medium was changed to eliminate dead cells. Colonies of the surviving cells were fixed, stained and counted by microscopy as described in (16). A549 cells were fixed and stained after ca. 7 days of incubation, Bj5Ta, Nci-H1650 and Nci-H1975 - 10 days, and Nci-H522 - ca. 12 days, respectively. A group of cells with at least 50 cells was defined as a colony. For each UNT cell-line, the plating efficiency (PE) was estimated as the ratio of “colonies number” to the number of seeded cells. The survival fraction (SF) is represented as a percentage of counted colonies per number of seeded cells divided by the PE. Cell survival is expressed as logarithms of the SF versus dose.

**Table 1:** Number of cells per well in colony formation assay.

Treatment day/cell line	Bj5Ta	A549	Nci-H522	Nci-H1650	Nci-H1975
UNT^1^ day 24 and 30	150	100	300	250	250
NormRT or HypoRT/ day 24 and 30	300	200	600	500	500

^1^untreated cells (UNT).

### Statistical evaluation

Statistical evaluations were performed in GraphPad Prism (version 9.0.0; GraphPad Software). For two groups’ comparisons, a Student’s t-test was applied. To compare three or more groups, one-way ANOVA was used. P values with α<0.05 were considered significant.

## Results

### Proliferation scale

**Figure 1:**
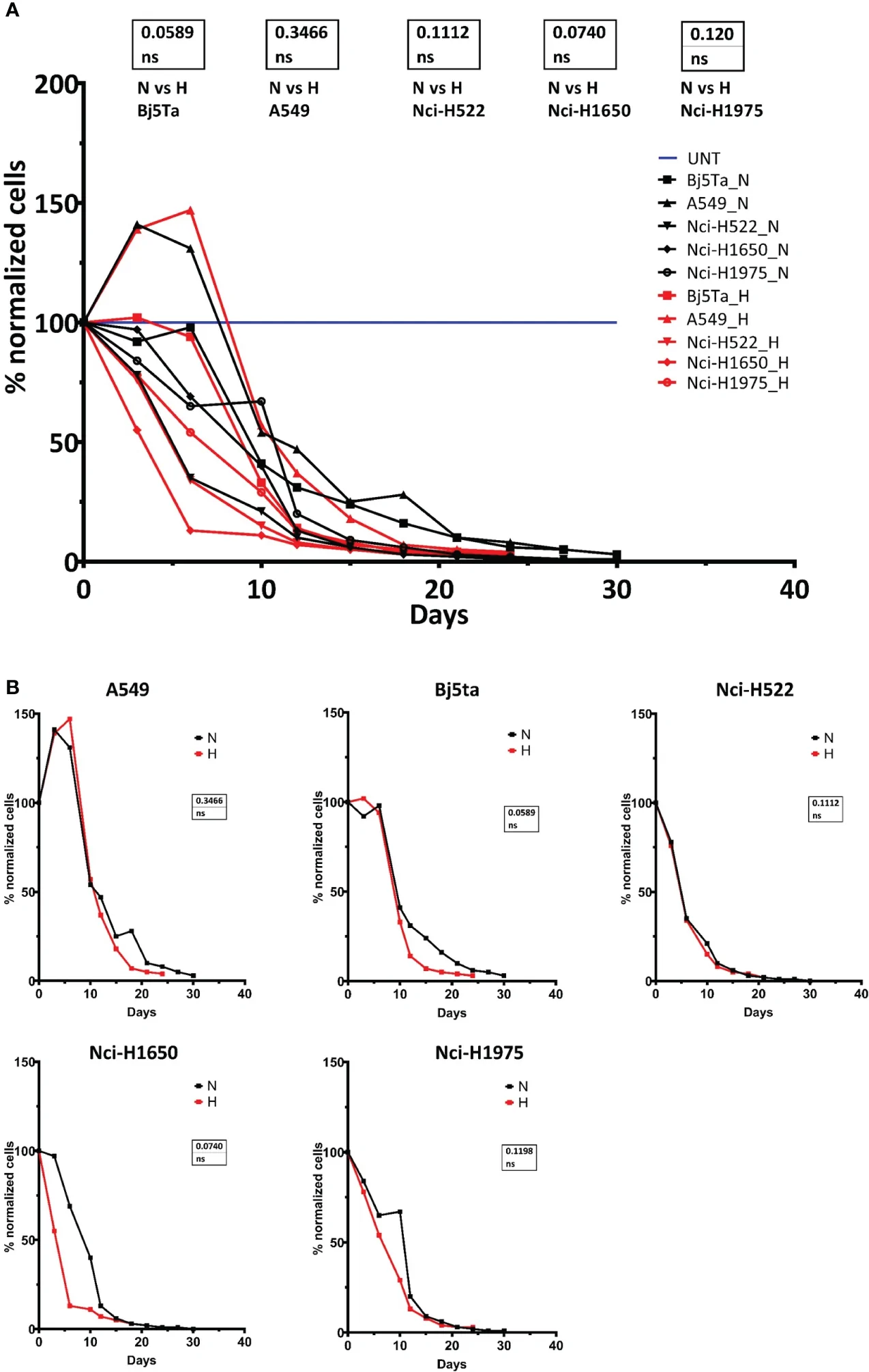
Cell proliferation capacity during RT. Counted cell numbers are plotted as a percentage after normalization to the number of cells seeded at the beginning of experimental line. Combined cell proliferation for all cell lines **(A)** and for each – individually **(B)**. “H” or red line represents HypoRT, “N” or black line represents NormRT, blue line represents UNT (untreated cells).

We investigated growth and proliferation capacity via the number of directly counted cells and by means of an MTT assay. In direct counts, we obtained a continuous reduction in the number of cells over observation time. The growth scale of A549 (for both irradiation treatments) was the highest among all cell-lines until ca. day 12, and afterwards, all the cells had a very similar proliferation scale (**Figure 1**). There was a slight but non-significant trend for Bj5Ta cells and Nci-H1650 (**Figure 1**) in HypoRT protocol as compared to NormRT.

**Figure 2:**
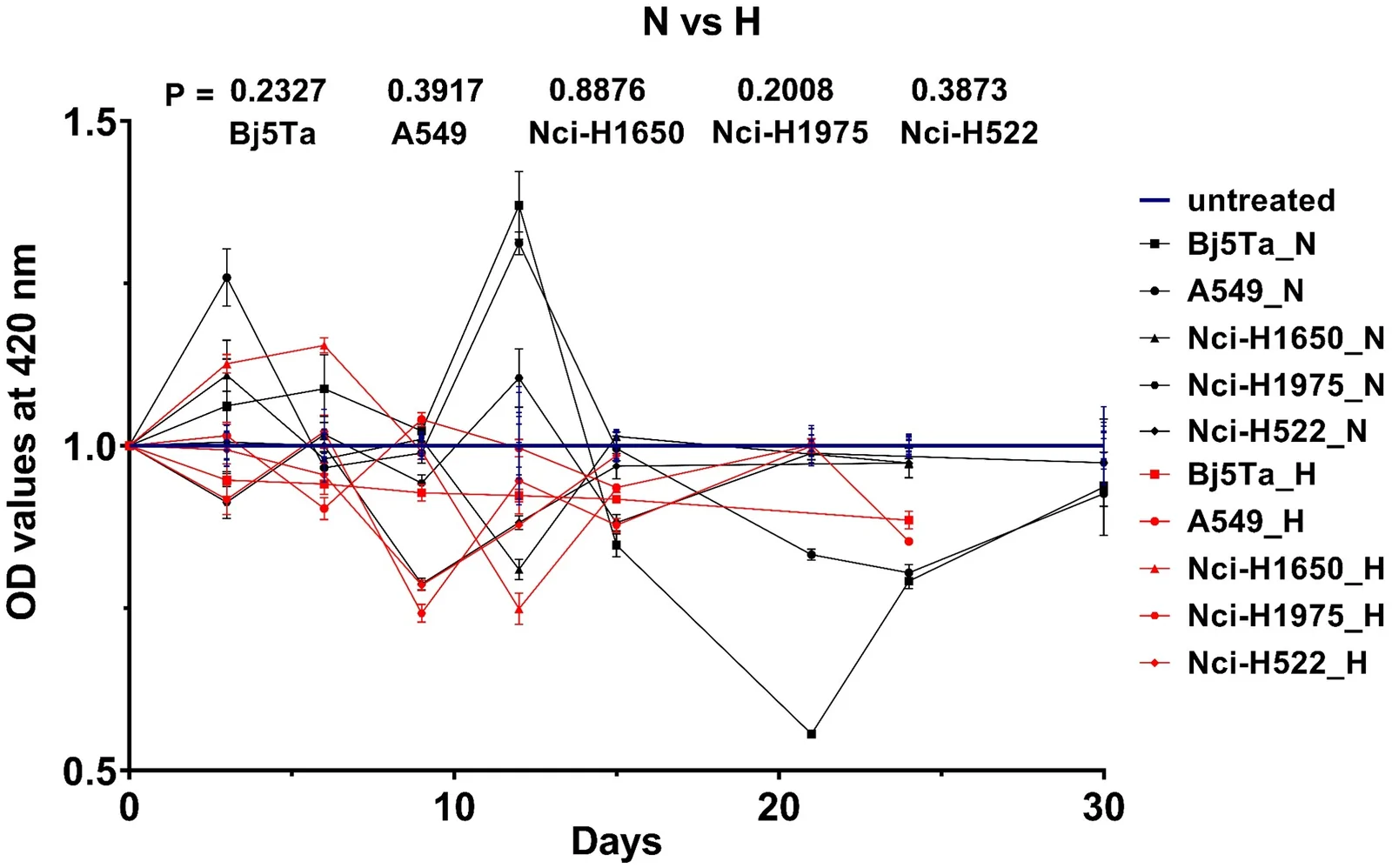
Cell growth rate measured by MTT assay during RT. OD values after normalization to the untreated estimates (UNT) are plotted versus irradiation day. UNT estimates of the individual cell lines were normalized to 1 und are represented by the blue lines. The black lines (or “N”) correspond to NormRT and red lines (or “H”) to HypoRT, respectively (vs – versus).

We found no significant difference in proliferation rates for any of the employed cell-lines using the MTT assay after HypoRT or NormRT protocols (**Figure 2**). In general, the cell-lines behaved differently until irradiation day 15, whereas afterwards, the growth rate of all the cells was continuously inhibited, with a pronounced effect seen in Bj5Ta at day 21 in the NormRT protocol (**Figure 2**).

**Figure 3:**
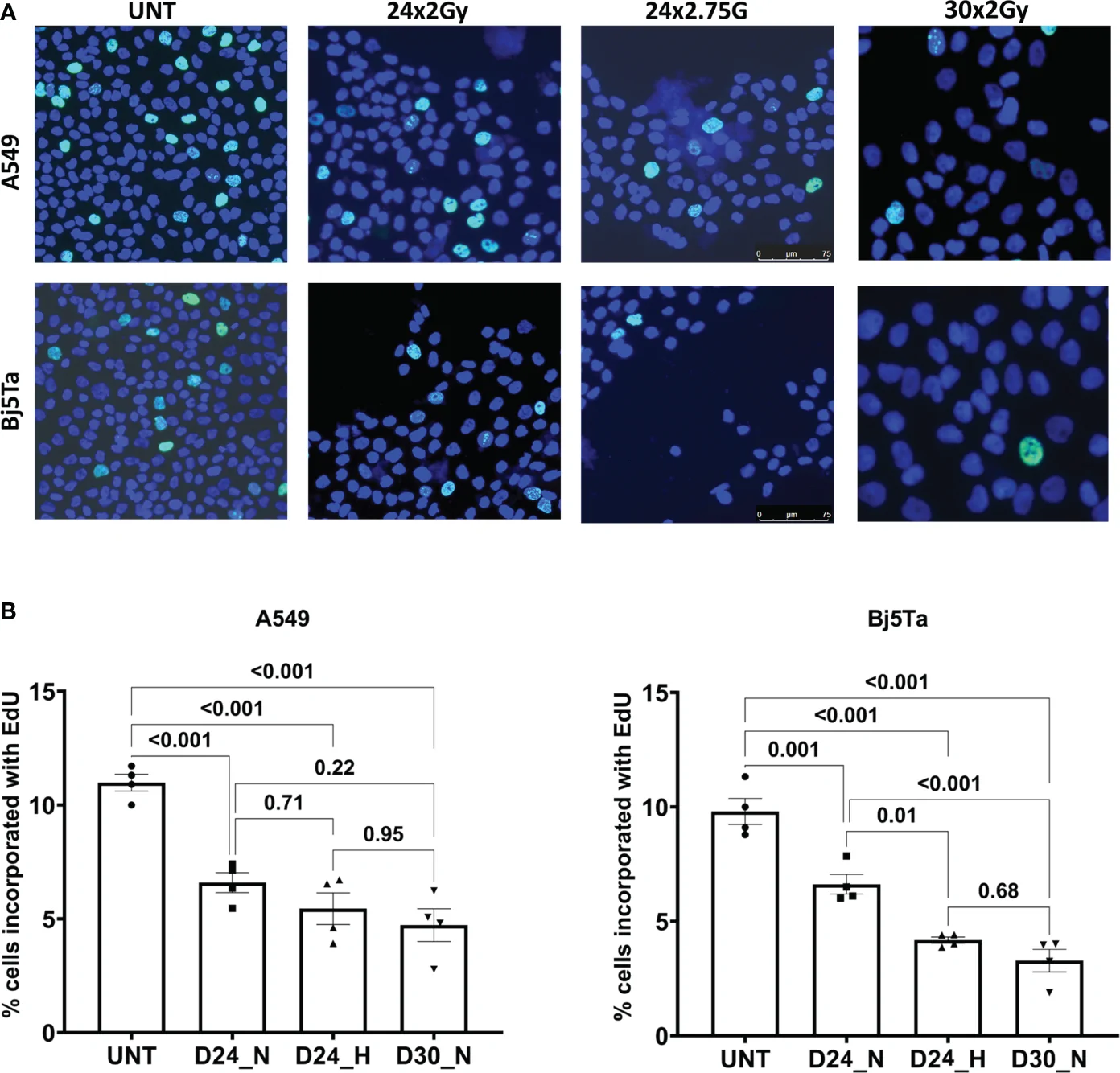
DNA synthesis–based cell proliferation (Bj5Ta and A549). Illustrative images of EdU stain in green **(A)** and corresponding evaluation of cell percentages on irradiation day 24 (D24) and irradiation day 30 (D30). **(B)**. DNA is counterstained with DAPI (blue). Images were taken with 20x objective under a Leica DMI6000B microscope. The percentage of EdU stained cells is expressed as a bar plot +/- SEM from technical duplicates with two evaluation regions. UNT represents untreated values; “N” corresponds to NormRT and “H” to HypoRT, respectively.

In the DNA synthesis–based cell proliferation (EdU incorporation assay) for A549 and Bj5Ta cells, we found a marked difference at day 24 between HypoRT and NormRT in Bj5Ta cells (**Figure 3A**), however, this effect was not significant for A549 cells (**Figure 3B**).

**Figure 4:**
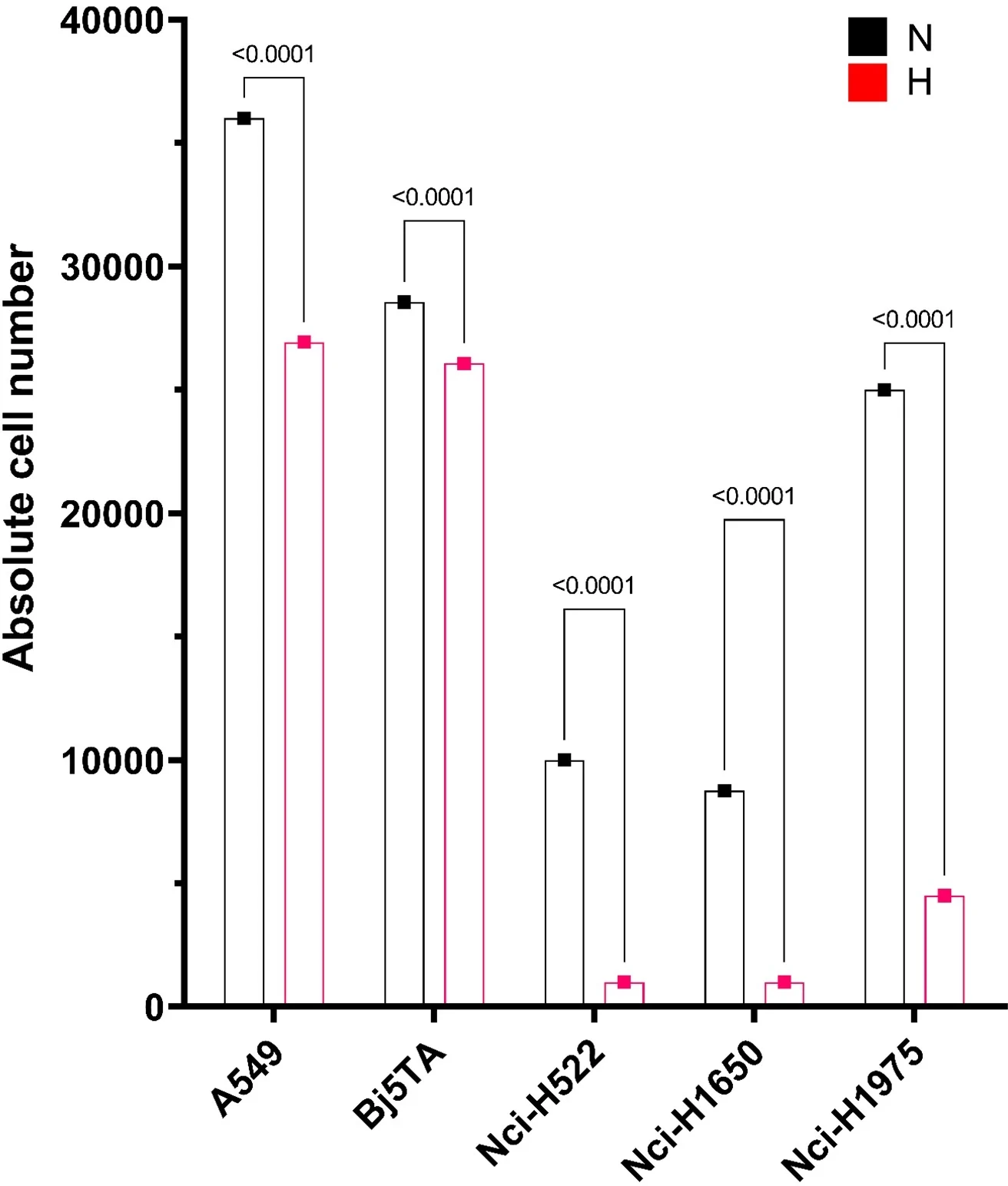
Absolute cell numbers by the end of the corresponding irradiation protocol. Cell numbers for every cell line employed are presented as an average, calculated from two combined scores, each includes two independent values of manual and automated counts. Absolute cell numbers are presented as a bar plot with red or “H” for HypoRT (day 24) and black or “N” for NormRT (day 30).

The Nci-H522 cell-line was the most IR sensitive in a proliferative assay, being equally sensitive to both RT regimes and showing rapidly slowed growth (**Figures 1**, **2**). The growth rates of both Nci-H1650 and Nci-H1975 cell-lines were also prominently slowed down at about irradiation day 20 (both irradiation regimes) and they behaved similarly to Nci-H522 in the HypoRT protocol (**Figure 1**). In contrast, A549 (being the most proliferative) and non-cancer cell-line Bj5Ta continued to proliferate with the daily exposure, albeit at a retarded rate. All the cell-lines started with 9x10^5^ cells and less than 5% of the seeded cells survived daily irradiation by the end of NormRT or HypoRT protocols (**Table 2**). When absolute cell numbers of this survival fraction were plotted against the irradiation protocol for every cell-line employed, HypoRT regime significantly differed from NormRT with a stronger effect (**Figure 4**).

**Table 2:** Total numbers of the cells counted at irradiation day 24 and day 30.

RT Protocol Day/Cell ID	Bj5Ta	A549	Nci-H522	Nci-H1650	Nci-H1975
NormRT_Day 24	5.2x10^4^	7.5x10^4^	1.3x10^4^	1.1x10^4^	3.6x10^4^
HypoRT_Day 24 (end)	2.6x10^4^	2.7x10^4^	1.0x10^3^	1.0x10^3^	5.0x10^3^
NormRT_Day 30 (end)	2.9x10^4^	3.6x10^4^	1.0x10^4^	8.7x10^3^	2.5x10^4^

### Efficiency of DNA DSB repair

**Figure 5:**
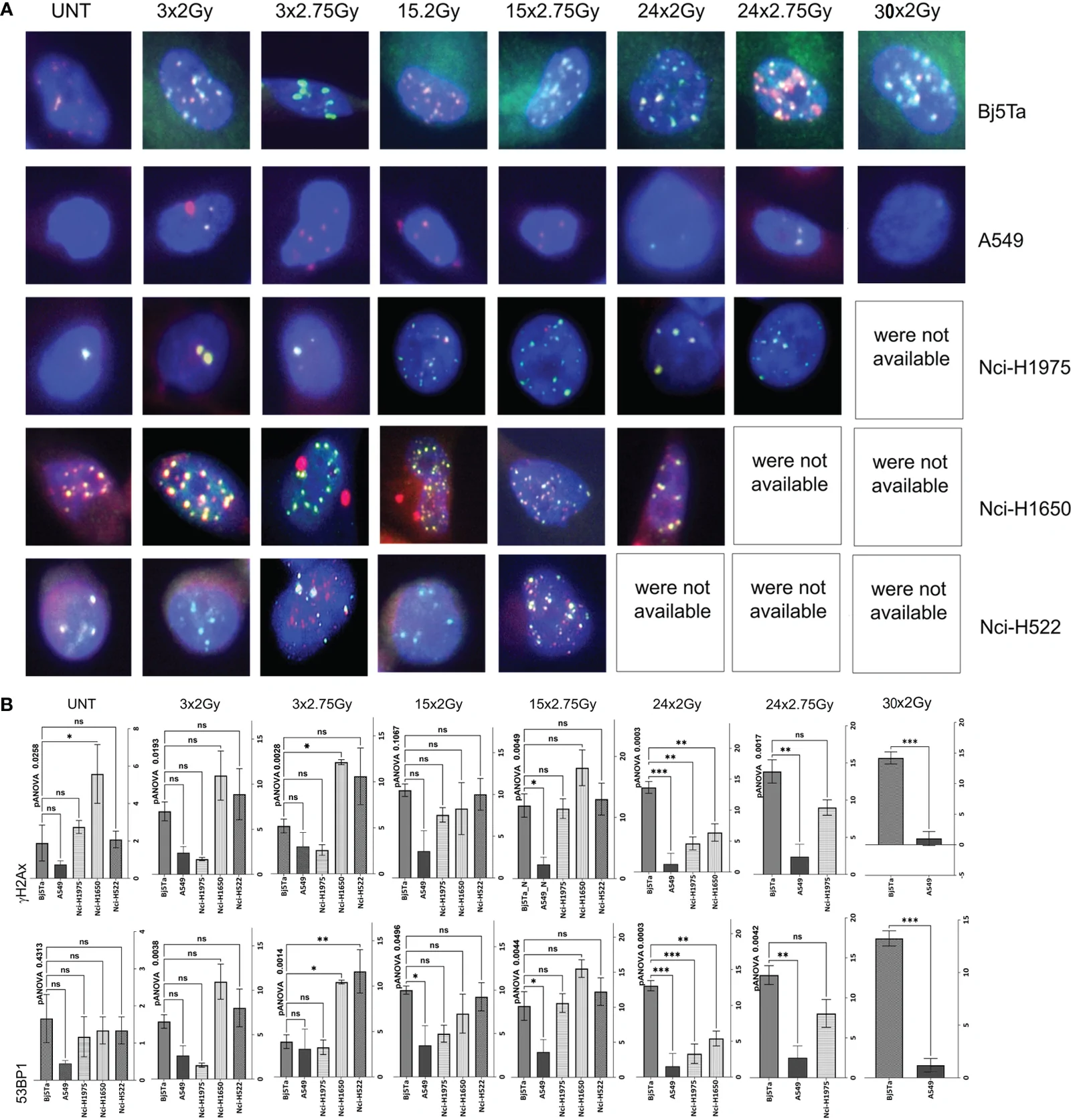
Analysis or residual DNA damage foci. Representative images of double staining for γH2AX foci in red or 53BP1 foci in green **(A)**. DNA is counterstained with DAPI (blue). Images were taken with 63x objective and a 1.6x artificial zoom under a Leica DMI6000B microscope. Evaluation of residual DNA damage foci with analysis for γH2AX on top and for 53BP1 on bottom **(B)**. Cells were fixed and stained 24h after IR with either HypoRT or NormRT, respectively, at days 3, 15,24 and 30 (NormRT only for A549 and Bj5Ta cells). Bar plots with +/- SEM represent an average numbers of residual DNA damage foci from technical duplicates (per cell and per slide) in different cancer cell lines compared to Bj5Ta cells. UNT corresponds to untreated values of “matched” controls at day 24, ns, non significant, *P < 0.05, **P < 0.01, ***P < 0.001.

To elucidate the involvement of DNA repair in survival of cells after the application of multiple IR fractions, we analysed residual γH2AX and 53BP1 foci during RT (**Figure 5**, **Table 3**). We observed no changes in the levels of residual foci after IR (both types) at any investigation day (**Table 3**) for two cell-lines: A549 (was the most resistant) and Nci-H522 (was the most sensitive) in other experimental settings. Moreover, no significant differences were found for both cell lines in responsiveness to different irradiation regimes, reflecting some resistance (A549) or sensitivity (Nci-H522) to different fraction doses. Nci-H522 could not be evaluated by means of residual foci at irradiation day 24, since 24h after irradiation, no viable cells were detected. The same was noted for Nci-H1650 after HypoRT (**Figure 5A**). In general, both Nci-H1650 and Nci-H1975 cells had significantly higher levels of residual foci after HypoRT (**Table 3**). For Bj5Ta cells, we observed a significant increment in residual γH2AX/53BP1 foci after day 15 (**Table 3**). There were noticeable differences in number of residual foci (both types) between cancer cell-lines, especially in contrast to the normal Bj5Ta cells (**Figure 5B**).Survival of the cells after conventional and hypofractionated irradiation

**Table 3:** DNA DSB repair efficiency after HypoRT and NormRT by means of residual foci.

Groups compared^1^	Bj5Ta		A549		Nci-H522		Nci-H1650		Nci-H1975	
	γH2Ax	53BP1	γH2Ax	53BP1	γH2Ax	53BP1	γH2Ax	53BP1	γH2Ax	53BP1
UNT ^2^ vs 3x2Gy	n.s	n.s	n.s	n.s	n.s	n.s	n.s	**	n.s	n.s
UNT vs 3x2.75Gy	n.s	n.s	n.s	n.s	n.s	n.s	n.s	**	n.s	n.s
UNT vs 15x2Gy	*	**	n.s	n.s	n.s	n.s	n.s	n.s	n.s	n.s
UNT vs 15x2.75Gy	***	**	n.s	n.s	n.s	n.s	*	***	***	***
UNT vs 24x2Gy	***	***	n.s	n.s	n.a	n.a	n.s	n.s	n.s	n.s
UNT vs 30x2Gy	***	***	n.s	n.s	n.a	n.a	n.a	n.a	n.a	n.a
UNT vs 24x2.75Gy	***	***	n.s	n.s	n.a	n.a	n.a	n.a	*	*
3x2Gy vs 3x2.75Gy	n.s	n.s	n.s	n.s	n.s	n.s	n.s	n.s	n.s	n.s
3x2Gy vs 15x2Gy	n.s	n.s	n.s	n.s	n.s	n.s	n.s	n.s	n.s	n.s
3x2Gy vs 24x2Gy	n.s	*	n.s	n.s	n.a	n.a	n.s	n.s	n.s	n.s
3x2.75Gy vs 15x2.75Gy	*	*	n.s	n.s	n.s	n.s	n.s	n.s	**	**
3x2.75Gy vs 24x2.75Gy	*	***	n.s	n.s	n.a	n.a	n.a	n.a	n.s	n.s
15x2Gy vs 15x2.75Gy	n.s	n.s	n.s	n.s	n.s	n.s	n.s	*	n.s	n.s
15x2Gy vs 24x2Gy	n.s	n.s	n.s	n.s	n.a	n.a	n.s	n.s	n.s	n.s
15x2.75Gy vs 24x2.75Gy	n.s	n.s	n.s	n.s	n.a	n.a	n.a	n.a	n.s	n.s
24x2Gy vs 24x2.75Gy	n.s	n.s	n.s	n.s	n.a	n.a	n.a	n.a	n.s	n.s
30x2Gy vs 15x2Gy	n.s	n.s	n.s	n.s	n.a	n.a	n.a	n.a	n.a	n.a
30x2Gy vs 24x2Gy	n.s	n.s	n.s	n.s	n.a	n.a	n.a	n.a	n.a	n.a

Results of one-way ANOVA with multiple statistical test correction. ^1^Groups compared represent normalized foci numbers to matched non-irradiated estimates for each of the tested groups, ^2^ Untreated cells (UNT), vs, versus, n.s, not significant, *P < 0.05, **P < 0.01, ***P < 0.001, n.a, not applicable.

**Figure 6:**
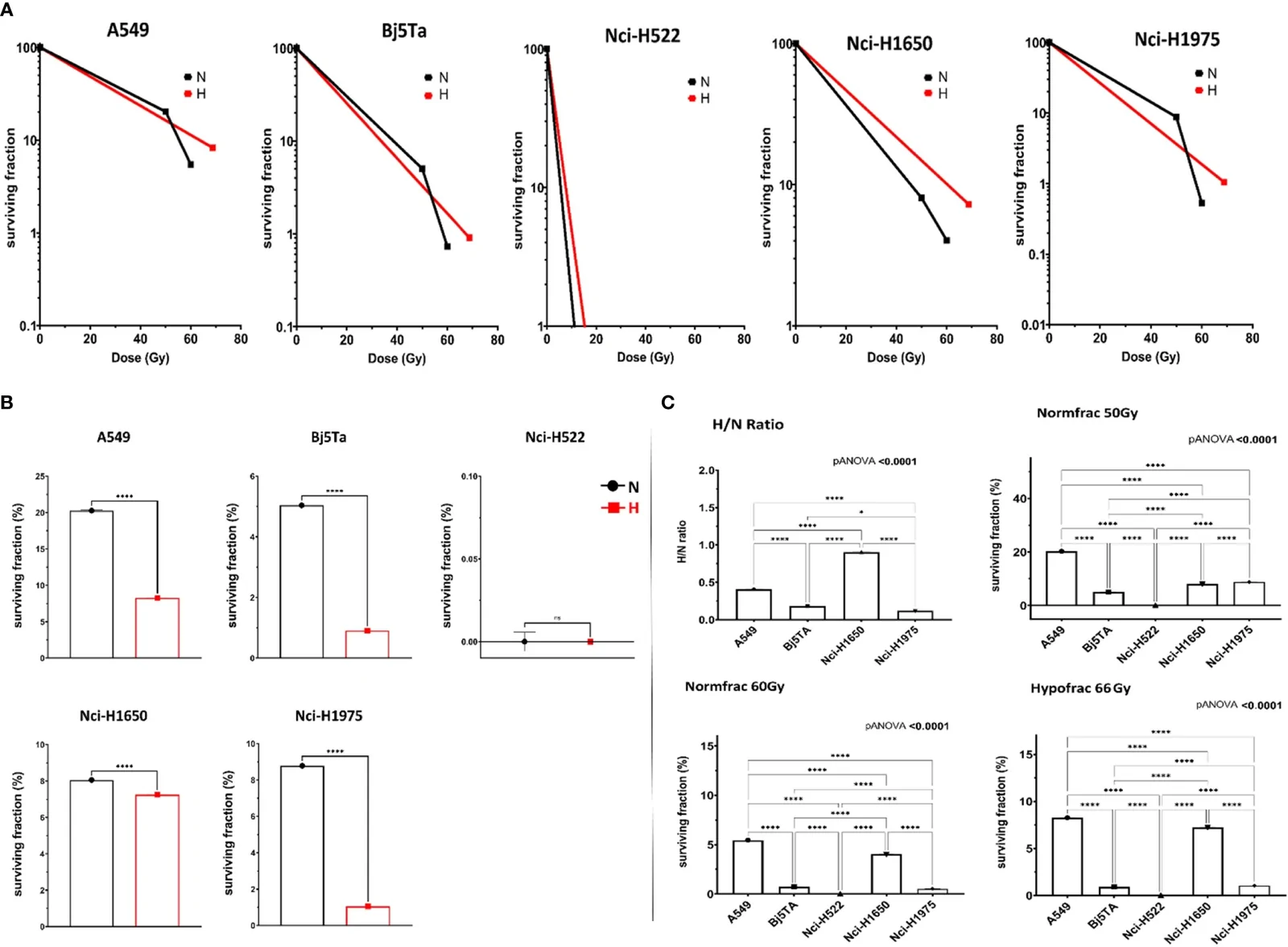
Cell survival after corresponding irradiation protocol. Clonogenic cell survival after irradiation with HypoRT (red line or “H”) and NormRT (black line or “N”) **(A)**. Efficacy of HypoRT (red or “H”) regime compared to NormRT (black or “N”) by the end of the treatment, defined as SF in % **(B)**. Ratio of HypoRT/NormRT **(C)**, top, left). Cell survival in this analysis was calculated as SF after cumulative dose of 66 Gy/SF after cumulative dose of 60 Gy. Efficacy of HypoRT in contrast to NormRT, is expressed as SF (percentage of cells at irradiation day 24/25 after cumulative dose of 66 Gy (HypoRT, “Hypofrac”) **(C)**, bottom, right) and 50 Gy (NormRT, “Normfrac”) **(C)**, top, right). Efficacy of NormRT at irradiation day 30 (“Normfrac”), presented as SF (% after cumulative dose of 60 Gy) **(C)**, bottom, left). n.s, not significant, *P< 0.05, ****P < 0.0001.

Plating efficiency in CFA assay was different in investigated cell-lines, with the lowest for Nci-H522 cells (about 6%) compared with 10% in Nci-H1650, 25% in Nci-H1975 cells, 55% in Bj5Ta or 65% for A549 cells, respectively. Nci-H522 was very slow in proliferating, whereas A549 was the quickest. After irradiation day 24, Nci-H522 turned out to be the most radiosensitive in a CSA, for both regimes, whereas other employed cells were more sensitive to the HypoRT schedule, with Nci-H1650 cells having comparable sensitivity to both protocols (**Figures 6A–C**). The reactions of the employed cells were distinct in CSA, indicating some heterogeneity and cell-line specificity.

## Discussion

Radiotherapy is a cornerstone in the treatment of NSCLC, especially for locally advanced tumors. Recent studies suggested no differences between NormRT and moderate HypoRT for patients who are ineligible for concurrent chemotherapy (12, 13). Therefore, HypoRT may be considered as a convenient alternative to NormRT, although some clinicians remain concerned about the efficacy and side effects. Generally, cells (tumor and normal) show improved survival after fractionated irradiation as compared to one single large irradiation dose (17). Thus, dose fractionation can reduce damage to normal non-malignant cells (mainly conventional NormRT with doses of 1.8-2.0 Gy per fraction) but low fraction doses can also limit the anti-tumor potency of IR, affecting the proliferation potential of tumor cells. Advances in RT accuracy omit the need for large target margins and the amount of normal tissue exposure is reduced, allowing an increase in the total-dose or total-dose efficacy.

In NSCLC treatment, the efficacy and toxicity of moderate HypoRT and Norm RT was proved in several randomized clinical trials with controversial outcomes. Comparable survival and side effects in slight favor of HypoRT, due to the shorter treatment duration and higher convenience for patients, were found in studies by Iyengar et al. and Zayed et al. (12, 13). Hughes et al. demonstrated the superiority of HypoRT in a small clinical trial (18). These studies of modest or moderate hypofractionation are fewer and mostly single arm or single institution with fewer participants. Parisi et al., in a large review-analysis, found that HypoRT is safe in patients with locally advanced NSCLC, but suggested that such radiation schemes need further investigation (19). In contrast, Brada et al. demonstrated the superiority of NormRT in an analysis of a comprehensive national radiotherapy data set of 169,863 cases (20). However, knowledge about cellular processes during radiotherapy, applied over time in different fractionation methods, is limited.

In systematic review articles, Robinson (21) and Kepka (22) summarized that an oncologic benefit for the use of HypoRT in NSCLC has so far only been postulated theoretically but has not yet been proven empirically or experimental. Understanding the mechanisms that lead to side effects and mechanisms, that assist cancer cells to survive after IR, can improve patient outcome, and help to identify new approaches for better treatment. There are a number of clinical studies, reporting on the efficiency of hypofractionation. Moderate hypofractionation has been shown to be non-inferior to normofractionated treatment and is strongly recommended by NCCN guidelines in the primary setting for low- and intermediate-risk prostate cancer (23–27). HypoRT also appears to be non-inferior compared to NormRT in thyroid cancer (28). Hypofractionation is suggested to be a safe and feasible therapeutic option for glioblastoma (29, 30), large brain metastases (31) or soft tissue sarcomas (32). Safety and efficacy of hypofractionated RT for breast cancer treatment was reported in a number of studies (33–36). However, to our best knowledge, there are very little preclinical experimental data on *in vitro* radiobiology of hypofractionation for any type of tumor, including our recent research on triple-negative breast cancer cell-line model (16). The bulk research on this subject deals with modelling radiobiological effects (37, 38). Direct comparison of fractionation schedules for NSCLC cell models in radiobiological approach in two recent studies reflects the clinical condition with some early benefits of hypofractionation in regard to a less-aggressive growth pattern (39, 40). Zhang et al. utilized just one fraction of 10 Gy (39) for HypoRT regime, which is much higher than any fraction used for the moderate hypofractionation. Wang et al. focused on establishing surviving/resistant cell lines by applying 20, 30 or 40 fractions of 2 Gy conventional dose (40). Since fractionation size may impact on radiation survival of different tumor cells, and thus on tumor resistance and recurrence (41), RT strategy optimization is evidently required. To address the issue of the benefit, local tumour control, and toxicity of HypoRT in NSCLC in radiobiological context, we comparatively investigated the response of tumour and normal cell lines to HypoRT and NormRT schedules. Cells were irradiated daily, imitating the clinical situation.

Since IR can directly impact on different cell functions by provoking DSBs, inhibition of cell proliferation, cell death, we investigated the proliferation rate of the cells, the efficiency of DSBs repair, and survival capacity in a combination of functional, cell-biological and molecular assays during irradiation with different schedules. Our results revealed differences in the receptivity to applied irradiation regimens in the investigated cells. By means of MTT assays, no significant difference between hypofractionated or conventional RT was found. Only after day 15, all the employed cells were continuously inhibited, with a pronounced effect for Bj5Ta at day 21, confirming to some extent the low α/β -ratio of fibroblasts in comparison to cancer cells. In cell proliferation assay (by means of direct cell counting), a constant reduction in cell numbers over the treatment time was obtained for both regimes, with a significant difference in the number of surviving cells for all the investigated cell-lines by the end of each irradiation protocol, with HypoRT being superior to NormRT. This effect was most pronounced for Nci-H522 and Nci-H1650 cells, reflecting their more radiosensitive phenotype in comparison to A549 cells. Enhanced radiosensitivity of Nci-H522 and Nci-H1650 cell-lines was also noticed in other experimental read-outs. While analysing the DNA DSB repair capacity, we found notable differences between the investigated cells with no values (no viable cells detected) at irradiation day 24 for Nci-H522 (both protocols) and Nci-H1650 cells (HypoRT). Nci-H1650 cells as well as Nci-H1975 cells exhibited higher levels of residual γH2AX/53BP1 foci after HypoRT, which could indicate higher sensitivity to this regime. Normal Bj5Ta cells revealed a significant increment in residual foci after irradiation day 15 (both regimes); whereas cancer cells noticeably tended to have lesser residual foci after irradiation day 15 (**Table 3**, **Figure 5**) with A549 being the most resistant with no elevated residual γH2Ax/53BP1 foci at any investigation day or dose. Additionally, in Bj5Ta cells we noticed raised levels of residual foci after day 3 of subsequent irradiation when compared to untreated cells, which most likely suggests the inability of cells to complete the repair of IR-induced DSBs prior to the next IR insult (**Table 3**).

Increased levels of residual foci at 24 hours after irradiation can indicate that cells have more severe DSBs and slower or less efficient DSBs repair. Such a pattern was observed in Bj5Ta, Nci-H1650 and Nci-H1975 cells. Whereas no changes or a reduction in foci numbers, in comparison to pre-treatment, may indicate a better recovery and radio resistance potential in the surviving cells (such as in A459) (42, 43). Our results suggest that the cells either adopted or had efficient repair, and may reflect the observations in the resistant A549 cells. On the other hand, an absence of cells with an elevated amount of residual foci, or a general absence of viable cells, could be the consequence of accumulated DNA damage and DSBs. Such DNA damage accumulation after irradiation triggers replication stress and overruns the repair capacity, and can lead to cell death with irreparable DSBs. This may reflect our findings in the Nci-H522 or Nci-H1650 cancer cell-lines, as the most IR sensitive in our experimental settings, since no viable cells were detected at irradiation day 24.

DNA synthesis–based cell proliferation assay revealed a highly significant difference between HypoRT and NormRT in Bj5Ta cells, however, the increase in inhibited proliferation by HypoRT regime was not significant in A549 cells. For NormRT, both cell-lines behaved comparably, confirming a slower rate of radiation induced apoptosis in fibroblasts, and confirming an α/β -ratio of NSCLC (A549) as high as 10, estimated from clinical studies (44). Applying HypoRT, the higher single doses only slightly enhanced this effect on A549 cancer cells. Such an effect of non-accelerated clinical remission for HypoRT is also known from other tumor entities (45).

CSA revealed that Nci-H522 cell-line is the most radiosensitive for both regimes, whereas other employed cells were more sensitive to HypoRT, with Nci-H1650 cells having comparable sensitivity to both HypoRT and NormRT. It is important to note that Nci-H1650 and Nci-H1975 harbor *EGFR* mutations, among others, (Nci-H1650 additionally has *TP53* mutations and is PTEN null), whereas Nci-H522, in comparison to the other analyzed NSCLC cell lines, “only” contains a *TP53* mutation (in codon 191). The A549 cells are wild-type for such common mutations in lung cancer and are known to be radioresistant. The effect of *EGFR* mutations (as in Nci-H1650 and Nci-H1975) on clinical response to radiation is not well known. There is some evidence claiming that the degree of radioresistance or radiosensitivity correlates with the EGFR expression status (46), but *EGFR* mutation status could not be an independent factor to predict radiation response in cells (47). Therapeutic agents such as tyrosine kinase inhibitors can target lung cancers, carrying *EGFR* mutations, but further studies are needed to assess the degree of possible cross-resistance with radiotherapy [reviewed in (47)]. Some multiple- receptor tyrosine kinases inhibitors have also shown an effect on EGRF-wild-type cells such as A459 (48). They can be of potential interest for cells with clinically relevant mutations, providing an intrinsic resistance to some EGFR inhibitors, as in Nci-H1975 or Nci-H1650 (49, 50). However, there is some evidence that a combination with irradiation kinase inhibitors can raise individual radiosensitivity of normal tissue (51). The effect of *TP53* mutations on radiosensitivity is a controversial topic. Cells with *TP53* mutations could generally be more sensitive to ionizing radiation (52), as also observed for Nci-H522 and Nci-H1650 in our study. However, there are numerous studies suggesting that *TP53* mutations may increase radiosensitivity, and others reporting that *TP53* mutations cause radioresistance [reviewed in (53)]. These differences could be correlated with the type of p53 mutation (54) and the genetic background of the cell lines. Some additional somatic events or epigenetic changes during long-term culture and daily irradiation exposure cannot be excluded. Such changes, perhaps also in important DNA damage repair genes, might modified the outcome to some extent and should be noted. Additionally, exposure of cancer cells to very extended fractionation regimes can select for a radioresistant cancer subpopulation with modified cellular processes in response to subsequent radiation exposure. We do not think that we were able to produce radioresistant cell-lines in our experimental settings, since the EdU results demonstrate that irradiated A459 cells were less proliferative by the end of both treatment schedules when compared to parental matched cells, indicating likely no selection for a radioresistant cell population, but this possibly cannot be fully excluded. It is also important to note that the survival effect differs between cell lines, very likely due to the fractionation schedule itself, particular due to different BED equivalents. Comparing the total equivalent doses, the hypofractionation cohort absorbed more dose than normofractionated, which might as well explain the differences in outcome. In clinical trials, an improvement in overall survival was found to be associated with higher BED [reviewed in (22)]. BED is commonly used for isoeffective dose calculations and (as a mathematical derivation of the exponential part of the LQ model) belongs to an effective tool in preclinical and clinical radiobiology. BED has direct relationship with the cell survival in fractionated radiation treatments. In this context, the basic LQ model describes the SF as a function of the defined radiation dose delivered in tissues characterized with α/β LQ parameters. Most malignant tumors (in our case, non-synchronized cell cultures) assemble a population of cells with heterogeneous radiosensitivity. This corresponds to a different linear quadratic survival relation for different doses. The ability to repair radiation damage also follows an exponential curve with increased effectiveness at a decreasing dose per fraction. Thus, the larger the difference between the fractions in different protocols, the smaller the α/β ratio becomes, increasing the damaging impact of large size fraction compared to the smaller one in a single exposures. During a lengthy treatment, cell survival within the whole population will not follow a linear quadratic equation, especially when incomplete repair is taken into account. When ionizing radiation interacts with living cells, different biological processes can occur: interaction with all cells or just a portion of the cells, which after the first fraction produces a number of “killed” and a number of sub-lethally damaged cells from the initially undamaged population. During the next successive fractions, the radiation can interact with all these kinds of cells and can produce the same “cell-types”: from undamaged cells and sub-lethally damaged to “killed”. Consequently, the α and β values, which represent irreparable lethal damage and sub-lethal damage, respectively, as well as the α/β ratio, will differ between single exposures and accumulated fractions. With repeated fractions of irradiation, the irreparable damage in cells may increase (resulting in an increased α value), and some repairable sub-lethal damage (β value) may be converted into irreparable damage (α value), increasing at an increasing dose per fraction, as in the HypoRT regime.

In general, if we rank the employed NSCLC cell-lines according to the radiosensitivity from highest to the lowest (regardless of the irradiation concept), Nci-H522 is the most sensitive, followed by Nci-H1650, Nci-H1975 and A549 as the most resistant, as observed in our experiments. In recent years, the concept of treatment strategy stratification based on tumor genetic profiles has become widespread, indicating that “mutational signature” affects cancer cell radiosensitivity as well. From common mutations in lung cancer, Nci-H522 harbors “only” a single base deletion in *TP53* at codon 191 (CCT→CT) with a reported LOH of TP53 locus (55). Additionally it has been reported that Nci-H522 has low levels of SDH5 (succinate dehydrogenase 5) and this SDH5 deficiency may contribute to “additional” radiosensitivity through TP53 axis (56). Nci-H1650 cell-line has *EGFR*, *TP53* and *PTEN*-null mutations, and the latter can contribute to the establishment of radiation resistance (57) and may have influenced the “intermediate” sensitivity of these cells in our experiments. Nci-H1975 cells with “only” an *EGFR* mutation seems to be less sensitive compared to Nci-H1650 in some read-outs, although both cell-lines have similar EGFR levels, but different mutations (58), which may impact radiation sensitivity differently. A549 cells harbor a single nucleotide mutation G12S in *KRAS* gene (very rare mutation for NSCLC), which leads to the substitution of a glycine for a serine and confers constitutive activation of KRAS (59). Such KRAS-activating mutations are oncogenic drivers and correlate with the known mutation-mediated radioresistance (60). Thus, radiosensitivity or radioresistance, as clinical tumor radioresponsiveness, maybe influenced by a wide range of genetic aberrations and requires genetic profiling.

Some limitations of the study need to be acknowledged. The use of cell models may partially limit some results, especially in regard to the *in vivo* situation, which may recapitulate the clinical scenario in a more efficient way. However, there is a causal link between clinical and cellular radiation reactions, and employment of well-established cell culture models in *in vitro* studies has an advantage that other groups can reproduce the experimental data. Considering the fluctuations in radiosensitivity between the different cell-lines and cell-types, utilisation of additional cell-models or some primary tumour material might provide a more comprehensive assessment of radiobiological dynamics after different irradiation regimes *in vitro*. It will also be interesting to see if the surviving cells are radically different at a molecular level to the parental cell-lines and can contribute to tumor radioresistance. Such clinically relevant resistance can affect the efficacy of RT, which is one of the most critical issues in therapy. Future possibilities include an investigation of the impact of different radio-chemotherapy combinations. *In vivo* models can contribute to a more complete elucidation of radiobiological effects. Nevertheless, the present data provide some framework for subsequent radiobiological studies.

Our combined observations indicate that responsiveness and sensitivity of all the investigated cells to IR vary in *in vitro* experiments. Although our findings in lung cancer cells and fibroblasts reveal almost similar benefit and toxicity of HypoRT and NormRT in *in vitro* settings, we provide some evidence for slightly favourable trend towards a HypoRT protocol. Identifying the suitable dosing scheme, as well as potential therapy combinations for any tumor entity may significantly affect patient survival and treatment outcome.

## Conclusions

Our findings showed approximately similar resistance and sensitivity to different fractionation doses in NSCLC, suggesting that moderate hypofractionation alone does not have the massive ablative effect of radiation, but has a certain superiority to NormRT. Therefore, protocols with accelerated dose per fraction (in combination with other therapeutic agents, based on the “mutational signature” of tumor) may open a therapeutic window for radiation dose optimization in NSCLC.

## Data availability statement

The original contributions presented in the study are included in the article/supplementary material. Further inquiries can be directed to the corresponding author.

## Author contributions

ML: Writing – review & editing, Validation, Methodology, Investigation. KK: Writing – review & editing, Validation, Methodology, Investigation. DR: Writing – review & editing, Visualization, Software, Formal analysis, Data curation. SG: Writing – review & editing, Validation, Methodology, Investigation. HC: Writing – review & editing, Resources, Project administration, Funding acquisition, Conceptualization. RM: Writing – review & editing, Supervision, Resources, Project administration, Conceptualization. CH: Writing – review & editing, Visualization, Supervision, Resources, Project administration, Funding acquisition, Formal analysis, Conceptualization. NB: Writing – original draft, Visualization, Supervision, Project administration, Methodology, Formal Analysis, Data curation, Conceptualization.
